# Greater similarity of conscientiousness scores in dyads is associated with greater interpersonal neural synchrony while completing a goal-oriented task: a brief report

**DOI:** 10.3389/fnhum.2025.1622203

**Published:** 2025-11-03

**Authors:** Vyara Stoyanova, Chris Ashwin, Chiara Scarampi, Muhammad Hijazy, Felix Carter, George Stothart, Neal Hinvest

**Affiliations:** ^1^Department of Psychology, University of Bath, Bath, United Kingdom; ^2^Centre for Applied Autism Research (CAAR), Department of Psychology, University of Bath, Bath, United Kingdom; ^3^Centre for the Interdisciplinary Study of Gerontology and Vulnerability (CIGEV), University of Geneva, Geneva, Switzerland; ^4^School of Professional Business Education, Faculty of Business and Law, University of Northampton, Northampton, United Kingdom

**Keywords:** personality, conscientiousness, openness, Big 5, neural synchrony, hyper-scanning, interbrain synchrony

## Abstract

Interpersonal neural synchrony provides a neural index of how individuals align cognitively and socially during interaction. While previous work has shown that personality traits shape interpersonal behavior, and that trait similarity can enhance dyadic coordination, little is known about whether such similarity predicts neural synchrony. The present study used an electroencephalography (EEG) hyper-scanning methodology to investigate the relationship between the degree of similarity in Big 5 scores of interacting participants in dyads and their interbrain synchrony during naturalistic dialogue. A total of 23 female dyads completed the Big 5 questionnaire and performed a goal-oriented social task while each wearing lightweight EEG headsets. Similarity for each Big 5 personality scale was created by calculating the absolute difference between the two participants within each dyad. Interpersonal neural synchrony was measured using Dynamic Time Warping (DTW), which quantified the similarity between separate temporal signals, based on a time-frequency decomposition of EEG. Results showed that similarity of Conscientiousness scores within dyads significantly predicted interpersonal neural synchrony within dyads (with openness showing marginal prediction). No relationship was evident for any other Big 5 trait. These findings demonstrate that personality similarity, particularly in conscientiousness, contributes to interpersonal neural synchrony, highlighting a trait-based pathway through which social alignment emerges during naturalistic interaction.

## Introduction

Humans perceive and navigate their social interactions in unique ways. For some individuals, social communication comes with feelings of excitement, while for others it brings distress and anxiety (Matz et al., 2022). Personality traits influence how people view and engage with each other and can predict people’s behavior during social interactions (Cuperman and Ickes, 2009). The Big 5 are some of the most well-known personality traits and includes extraversion, neuroticism, openness, agreeableness and conscientiousness (Carducci, 2009). According to McCrae and Costa (2003), extraversion characterizes how talkative and assertive a person is; neuroticism marks one’s tendency to worry and be self-conscious and emotionally vulnerable; openness highlights a person’s degree of imagination, creativity, open-mindedness, and curiosity; agreeableness is one’s ability to be empathic, trusting and soft-hearted; and conscientiousness is characterized by being hard-working, ambitious, organized and self-disciplined toward goals.

Research has reported that all Big 5 personality dimensions are significantly correlated with group organizational performance (Aremu et al., 2018). Specifically, teams consisting of moderately extroverted but highly open to experience and conscientious individuals, perform better on tasks involving creativity and idea-generation (Buchanan, 1998). Exhibiting neurotic personality traits (i.e., being anxious over one’s business performance) has also been linked to increased innovation of new products, increased sales and stock value among small businesses (Jawabri, 2020). According to several meta-analyses and systematic reviews conscientiousness generally shows the strongest relationship with performance (Hurtz and Donovan, 2000; Mount et al., 1998; Salgado, 1997). Findings from research have demonstrated that greater conscientiousness within a team is associated with better team performance including decision-making (Barrick et al., 1998; Neuman et al., 1999; Neuman and Wright, 1999; Peeters et al., 2006; van Vianen and De Dreu, 2001). Neal et al. (2012) looked at the relationship between the Big 5 traits and individual, team and organization performance in a large sample of Australian government employees and supervisors. Results showed that conscientiousness was positively related to both individual and team performance across all the measures included, while neuroticism was negatively related to the individual and team performance across measures. Research has reported that conscientiousness is associated with team cooperation (LePine and Van Dyne, 2001), with closer Conscientiousness scores between group members being related to better team performance (Kichuk and Wiesner, 1997). Based on the research it has been proposed that similarity in conscientiousness leads to cohesion between team members while dissimilarity in conscientiousness and extroversion may lead to conflict and reduce a team’s effectiveness and satisfaction (Mohammed and Angell, 2003; Molleman et al., 2004; Peeters et al., 2006; van Vianen and De Dreu, 2001). While relationships have been shown between conscientiousness and group performance at the behavioral level, there has been little research reported to date about the neural underpinnings of relationships between personality traits during group performance.

The emergence of hyper-scanning techniques has allowed researchers to investigate brain activity during group activities with two or more individuals (Valencia and Froese, 2020), often showing greater neural synchrony during interpersonal interactions. For example, research has shown that people’s brain activity synchronizes when they interact with each other verbally or doing motor activities including finger tapping or guitar playing (Sänger et al., 2012; Novembre et al., 2017). Dikker et al. (2017) used electroencephalography (EEG) to examine if the interpersonal neural synchrony in a group of students during their daily classroom activities related to personality factors. Results showed greater interpersonal neural synchrony in the students was related to greater social closeness, group affinity and empathic disposition scores, suggesting that neural synchrony in a group can provide a marker that predicts group dynamics. A study by Reinero et al. (2021) divided participants in groups of four and asked them to complete several problem-solving tasks, and teams with elevated interpersonal neural synchrony performed better than teams with lower interpersonal neural synchrony, while the level of interpersonal neural synchrony between individuals within control groups did not predict task performance.

Foundational theories of social interaction posit that interpersonal dynamics emerge from a continuous interplay between synchronization and segregation, reflecting inter- and intra-personal processes respectively (Mayo and Gordon, 2020; Froese et al., 2024). Periods of synchronization index shared attention, joint prediction, and the alignment of cognitive and affective states, while segregation reflects the maintenance of individual perspectives and self-regulation within interaction. Recent empirical work supports this framework. Hinvest et al. (2025) demonstrated that neural synchrony during naturalistic dyadic conversation was associated with the emergence of a shared social identity between partners. Taken together, these accounts provide a theoretical basis for understanding interpersonal neural synchrony as a dynamic marker of social alignment, whereby synchronization reflects the temporary convergence of individual minds into a shared representational space that supports joint understanding and coordinated action.

Some studies have closely examined the effect of personality traits on social synchronization, with some investigating the relationship between similarity in personality scores and interpersonal neural synchrony. For example, Cuperman and Ickes (2009) found that partners in dyads interact more effectively with each other when both are either introverts or extroverts, but not when their personalities mismatch. Highly agreeable partners were more likely to sit in closer proximity to each other, while highly conscientious ones held more frequent and longer eye contact. A study by Matz et al. (2022) grouped participants into dyads based on their personality traits and asked subjects to individually observe a set of images while undergoing EEG scanning to record dyadic interpersonal neural synchrony. Results indicated that the similarity in personality traits was a significant predictor of interpersonal neural synchrony between dyad partners. However, the dyads were not actually interacting with each other during the testing which limits the understanding about how Big 5 traits relate to neural synchrony while people engage socially with others.

Zhang et al. (2021) investigated neural synchrony in specific frontal regions of the brain measured by functional near infrared spectroscopy (fNIRS) while partners in dyads completed trials of a prisoner’s dilemma decision-making game, both individually and collaboratively. The researchers found greater interpersonal neural synchrony in the right inferior frontal gyrus (IFG) during group play, but not during individual play. Further results revealed that Extroversion and Agreeableness correlated with interpersonal neural synchrony during collaborative decision-making. The paradigm used focused on decision-making and only involved minimal, non-verbal, interaction between the participants. Using more naturalistic tasks where participants have greater and longer interaction with each other (including verbal communication) during a goal-oriented task with more global measures of interpersonal neural synchrony between the interacting participants may provide a better test of the relationship between the similarity of Big 5 personality traits and the neural synchrony of interacting participants.

Foundational theories propose that interpersonal synchrony provides a mechanism through which individuals achieve shared attention, prediction, and alignment during interaction (Sebanz et al., 2006; Hasson et al., 2012). Such alignment is not only transient but also socially meaningful, supporting processes such as the formation of shared identity (Hinvest et al., 2025). Personality psychology offers a complementary perspective, with the Big Five framework describing stable traits that influence regulation, flexibility, and social orientation (McCrae and Costa, 1999; Roberts et al., 2009). Evidence suggests that similarity between partners’ traits may facilitate compatibility in interactional norms, thereby enhancing synchrony (Arellano-Véliz et al., 2025). The current study therefore investigated the relationship between Big Five personality traits and interpersonal neural synchrony during naturalistic social interaction. Participants first completed Big Five questionnaires and were then paired into dyads to complete a goal-oriented discussion task on a topical societal issue while their neural activity was measured using wireless EEG. This design allowed us to test whether dyadic personality similarity predicts interpersonal neural synchrony during real-world conversation.

## Method and materials

### Participants

A total of 60 adult females were recruited for the study (mean age = 30.90, *SD* = 13.99). All participants were 18 years of age or older and spoke English as first language, and no participant reported history of psychiatric disorders or neurological trauma. Only females were recruited to control for sex differences in neural activation associated with social processing (Proverbio, 2021; Sato, 2020). Subjects were grouped into 30 dyads to complete the experiment, with each dyad consisting of females who were unacquainted with each other. The data for six of the pairs were removed because of technical problems with data collection, and one pair had to be excluded from the analysis because a participant’s age at the time of testing was outside the age range for this study. This left a total of 23 dyads which included 46 participants who were included in the final dataset for the study (mean age = 28.7, *SD* = 12.1).

### Materials

#### NEO-FFI personality inventory-30

The NEO-FFI-30 Personality Inventory was used to measure the Big 5 personality traits including neuroticism, extraversion, openness, agreeableness, and conscientiousness (Körner et al., 2008). The questionnaire includes 30 items in total, grouped into six items for each of the five traits. The items include statements and participants rate how much they agree with each statement on a 5-point Likert scale ranging from 1 (strongly disagree) to 5 (strongly agree). Internal consistency was evaluated using Cronbach’s alpha, with coefficients indicating primarily acceptable reliability: Agreeableness (α = 0.42), Openness (α = 0.59), Conscientiousness (α = 0.72), Neuroticism (α = 0.74), and Extraversion (α = 0.80). The NEO-FFI-30 has been validated in both German and Spanish samples, demonstrating reliability across cultural contexts (Körner et al., 2015; Fumero and De Miguel, 2023).

### Procedure

All participants provided informed consent to take part, and the study was approved by the Psychology Research Ethics Committee (PREC) of the university where the project was carried out. Each participant was randomly allocated to a dyad before commencing the experiment to ensure that dyad partners were unacquainted with each other. Each participant initially read an article about the plight of refugees on their own, and then during the testing session dyad partners had to discuss this topic together while EEG was acquired from both. Each participant was fitted with an Emotiv 14 channel wireless EEG headset (emotiv.com) 14 channel wireless EEG systems to measure EEG signals during the experiment from channels AF3, F7, F3, FC5, T7, P7, O1, O2, P8, T8, FC6, F4, F8, and AF4 (See Supplementary material for full details of the EEG methodology). Participants were first seated separately facing the wall and instructed to speak for a minute about a non-emotional topic, which served as a neutral control condition for the data analysis. The subjects were then seated facing each other at a table and engaged in a 10-min discussion about the key issues of immigration in the UK with the goal to come to a consensus agreement about how to resolve the problem. The 10-min dyadic discussion on a topical issue follows a well-structured paradigm, where short, structured but open-ended discussions are used to elicit naturalistic, yet comparable, exchanges across dyads. Discussion relevance was monitored *post hoc* by two members of the research team who agreed that all groups discussed relevant topics for the full conversation duration. Testing took place in a purpose-built observation laboratory with the researcher behind one-way glass, unobservable by the participants. The discussion took place in a social observation lab. The discussion was audio and video taped using four cameras situated at 90*^o^* angles directed at the dyads. After the researcher provided instructions, answered any questions, began recording and left the room, they sat hidden to the participants behind a one-way mirror. After completing the discussion, participants then separately completed questionnaires which are reported in a separate study (Hinvest et al., 2025). Finally, participants were debriefed and received £10 compensation for their participation.

### Data

Dynamic time warping (DTW) values representing the degree of interpersonal neural synchrony was the dependent variable while Big 5 Similarity scores to measure the degree of similarity in personality scores between participants in each dyad were the independent variable. DTW allows for most optimal alignment of two signals by iteratively warping the comparison path between their dyadic time-series data. Throughout dyads’ discussions, multiple DTW values were calculated, which were then averaged to create a single DTW score for each dyad. Lower DTW scores represent greater interpersonal neural synchrony (for more information on EEG acquisition and preprocessing and how the DTW measures were produced see the Supplementary material. Readers can also see our earlier work, Hinvest et al., 2025).

Big 5 “Similarity scores” were calculated for all five of the personality scales (i.e., Neuroticism, Extraversion, Openness, Agreeableness, and Conscientiousness), to measure the degree of similarity in the personality scores for each dyad. Similarity scores were created by calculating the absolute difference between the personality scores for the two participants within each dyad, with smaller scores representing greater similarity in personality scores between dyad members.

Outlier values for both DVs were detected using the Interquartile Range (IQR) method. Approximately 3.5% of the DTW data points fell outside the lower and upper bounds of the IQR and were excluded from the dataset before the DTW average scores were computed. There were no outliers among the Neuroticism, Agreeableness and Openness similarity scores, while Extraversion similarity had one dyad outlier and Conscientiousness similarity had four. Outliers in the personality measures were not excluded, as their removal would have substantially reduced the sample size and the statistical power of the study. After removal of DTW outliers, the DTW data was normally distributed (*W* = 0.95, *p* = 0.25). Big 5 Similarity scores for Neuroticism and Openness were both normally distributed (*W* = 0.94–0.96, *p* = 0.16–0.40), while scores were not normally distributed for Extraversion, Agreeableness and Conscientiousness (*W* = 0.85–0.90, *p* < 0.05). However, these distributions had skewness values of 1.46 for Extraversion, 0.68 for Agreeableness and 1.7 for Conscientiousness which are all deemed as acceptable (Hair et al., 2022) and a parametric statistical test was still considered suitable for the analysis.

## Results

A backward linear regression was used to identify possible predictors of dyad interpersonal brain synchrony (DTW average scores) out of all variables showing similarities in: Neuroticism, Extroversion, Agreeableness, Openness and Conscientiousness scores. The criteria for variable removal at each step was set at *p* > 0.100, and changes in Akaike Information Criterion (AIC) were cross-referenced to ensure model parsimony. No multicollinearity was detected, but as residuals were non-normally distributed and data appeared slightly heteroscedastic, we conducted a bootstrapping procedure with 5000 replications, and bias-corrected and accelerated 95% confidence intervals (BCa CIs) are reported below (Hayes, 2013).

**FIGURE 1:**
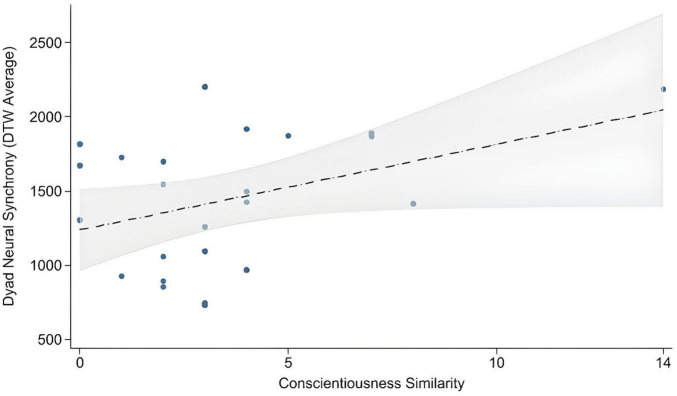
Scatterplot with regression line showing the association between dyad interpersonal neural synchrony and Conscientiousness similarity. Shaded area represents 95% Confidence Intervals.

Starting with the full model (M_0_) containing all five predictors returned non-significant results, *F*(5,17) = 2.19, *p* = 0.103, *R*^2^ = 0.39, Adjusted *R*^2^ = 0.21, *f*^2^ = 0.64, AIC = 348.79. The Similarity scores for Neuroticism (*p* = 0.468) were removed in the next model (M_1_), as their *p* values exceeded.100. Although the AIC of M_1_ improved to 347.49, the model remained non-significant *F*(4,18) = 2.68, *p* = 0.065, *R*^2^ = 0.37, Adjusted *R*^2^ = 0.23, *f*^2^ = 0.59. Statistical significance was first detected after Extroversion Similarity scores (*p* = 0.294) were further removed in M_2_, which had the Similarity scores for Openness, Agreeableness and Consciousness included as predictors *F*(3,19) = 3.30, *p* = 0.043, *R*^2^ = 0.34, Adjusted *R*^2^ = 0.24, *f*^2^ = 0.52, AIC = 346.60. The Similarity score for Conscientiousness was, however, the only individual predictor variable that significantly (i.e., *p* < 0.05) related to the prediction of interpersonal brain synchrony while the Similarity scores for Openness and Agreeableness were not significant (see Table 1). After further removing Agreeableness Similarity (as *p* value exceeded.100), the new model (M_3_) with only Openness and Conscientiousness Similarity as predictors remained significant *F*(2,20) = 4.54, *p* = 0.024, *R*^2^ = 0.31, Adjusted *R*^2^ = 0.24, *f*^2^ = 0.45 and provided a better fit than the full five-predictor model, with higher adjusted *R*^2^ (0.24 vs 0.21) and lower AIC (345.63 vs 348.79; ΔAIC = 3.16). Once again, Conscientiousness Similarity was the only individual significant predictor variable (see Table 1). When Openness Similarity was removed, leaving Conscientiousness Similarity as a single predictor (M_4_), the overall model returned a significant omnibus test *F*(1, 21) = 4.68, *p* = 0.042, *R*^2^ = 0.18, Adjusted *R*^2^ = 0.14, *f*^2^ = 0.22, AIC = 347.61), and examining the regression coefficients and bootstrapped confidence intervals revealed that Conscientiousness Similarity was a marginally significant predictor (see Table 1). The relationship between Conscientiousness Similarity and neural synchrony can be observed in Figure 1.

**TABLE 1:** Regression coefficients of statistically significant models.

							95% CI	
Model	Predictors	*SE*	*β*	*sr* ^2^	*t*	*p*	Lower	Upper
M_2_	(Intercept)	194.68	–	–	5.32	<0.001	689.67	1458.75
	Openness similarity	38.04	0.37	0.13	1.97	0.063	−7.68	141.73
	Agreeableness similarity	38.74	−0.18	0.03	−0.94	0.337	−106.82	46.20
	Conscientiousness similarity	26.37	0.51	0.25	2.69	0.033	9.19	116.04
M_3_	(Intercept)	177.31	–	–	5.37	<0.001	662.48	1362.45
	Openness similarity	35.40	0.37	0.13	1.94	0.062	−5.28	137.92
	Conscientiousness similarity	24.99	0.49	0.23	2.61	0.027	12.20	112.82
M_4_	(Intercept)	137.84	–	–	8.73	<0.001	917.87	1457.81
	Conscientiousness similarity	27.02	0.43	0.18	2.16	0.058	−2.72	109.35

Reported are bias-corrected and accelerated (BCa) 95% confidence intervals and bootstrap *p*-values based on 5000 replications.

## Discussion

Within our regression, similarity scores in Conscientiousness were the only individual personality factor that significantly predicted greater neural synchrony within dyads in our final model. None of the remaining Big 5 traits showed a significant contribution when examined as individual predictors.

Similarity in personality profiles has been linked to synchronized neural activity in passive viewing paradigms (Lahnakoski et al., 2014; Matz et al., 2022), an effect thought to arise from shared psychological perspectives during stimulus processing (Lahnakoski et al., 2014). The present work extends this line of research by examining whether specific personality traits predict neural synchrony during a goal-oriented, naturalistic, conversational task. This result aligns with evidence that conscientious individuals regulate attention and behavior in structured, predictable ways (Roberts et al., 2009), and that similarity in personality traits, particularly conscientiousness, enhances team effectiveness (Marjanović et al., 2023; LePine et al., 2011). These findings suggest that when both members of a dyad are similarly conscientious, they are more likely to establish compatible goals and behaviors (McCrae and Costa, 1999), yielding predictable temporal dynamics, consistent with joint action frameworks that emphasize the role of predictability in enabling alignment behaviorally (Sebanz et al., 2006) and neurobiologically (Hasson et al., 2012). This predictability facilitates mutual prediction and shared control states, core ingredients for inter-brain alignment in natural interaction (Dumas et al., 2010; Hasson et al., 2012). The present finding helps establish a brain-behavior link showing that dyads sharing similar degrees of conscientiousness show greater neural synchrony between them when completing a goal-oriented task together that is likely to reflect shared thinking and behaviors.

Although similarity in Openness did not reach conventional significance in predicting INS, the trend we observed is consistent with prior work linking openness (and its facets) to neural synchrony (Lim et al., 2024; Matz et al., 2022) and to activity within regions associated with the default mode network, a systems associated with self-referential processing and social perspective-taking (Rayá et al., 2023; Mulders et al., 2018). Openness is associated with cognitive flexibility, associative thinking, and a propensity to engage with novel or abstract ideas (McCrae and Costa, 1999). In a naturalistic conversational setting such as ours, these qualities may facilitate occasional alignment, especially when topics provoke evaluative or imaginative responses. However, because openness also entails variability in topic exploration and shifts in semantic framing, the temporal alignment necessary for robust neural synchrony may be less stable, leading to a weaker or trend-level effect rather than a strong one.

Results showed that the similarity scores for the remaining Big 5 personality trait scores within the dyads were not related to their interpersonal neural synchrony, which may be due to the characteristics of other personality traits being less involved in the goal directed task the participants worked together on in the current study. For example, highly agreeable people tend to be more soft-hearted and generous (McCrae and Costa, 2003), but the current goal-oriented task may not require these particular traits when coming to a consensus solution to a tough societal dilemma with the other person in the dyad.

Previous investigation of personality predictors of interpersonal neural synchrony within dyads during completion of a Prisoner’s Dilemma game found extroversion and agreeableness to be significant predictors of interpersonal neural synchrony, but not conscientiousness (Zhang et al., 2021). This difference likely reflects both methodological and theoretical factors. Methodologically, Zhang et al. (2021) restricted communication to brief non-verbal gestures within each trial, whereas our task involved extended, naturalistic verbal conversation. They also modeled synchrony in relation to individual-level Big Five scores, rather than dyadic similarity, and focused only on frontal EEG signals. Theoretically, these differences map onto the social functions of the traits themselves. Agreeableness, with its emphasis on pro-sociality and conflict avoidance (Jensen-Campbell and Graziano, 2001), may be particularly relevant in constrained, competitive–cooperative tasks where coordination is achieved through rapid alignment on cooperative strategies. By contrast, conscientiousness, associated with impulse control, order, and predictability (Roberts et al., 2009), is more relevant in extended, conversational settings where dyads must regulate turn-taking, sustain attention, and negotiate perspectives over time. Thus, while agreeableness facilitates synchrony in highly structured cooperative games, conscientiousness similarity fosters synchrony in open-ended dialogue by supporting compatible conversational norms and predictable temporal dynamics.

While the current experiment facilitated naturalistic face-to-face interactions to study human social engagement in a more ecologically valid setting, the design was still carried out with female-only dyads in a lab setting which is different to many social interactions in real-life which can occur in varied settings and with larger groups. Therefore, future research should include larger, more demographically diverse, samples engaging socially in various natural settings doing a range of tasks to investigate brain-behavior relationships. Potential covariates influencing neural synchrony, such turn-taking frequency, eye contact duration and time taken to reach consensus should also be addressed in future research.

The present findings suggest that similarity in Conscientiousness supports greater interpersonal neural synchrony during naturalistic conversation. This has potential implications for contexts where coordination and mutual understanding are critical. In education and workplace collaboration, matching individuals with similar conscientiousness may foster smoother dialogue and more efficient joint problem-solving. In therapeutic or clinical settings, greater trait similarity could strengthen rapport and engagement, whereas structured turn-taking strategies may help compensate when partners differ markedly in conscientiousness. More broadly, these results point to the importance of considering dyadic personality similarity, rather than only individual traits, when designing interventions or communication contexts that rely on alignment and cooperation.

In conclusion, the present study revealed novel findings about brain-behavior relationships in social neuroscience where higher similarity of Conscientiousness scores between participants in dyads was related to greater interpersonal neural synchrony between them while they complete a goal-oriented task. This effect was not seen for the other Big 5 personality traits, suggesting that similar Conscientiousness scores between dyad members was associated with key behaviors involving hard-work and striving toward a goal using cooperation that may underlie the higher neural synchrony. These findings advance our understanding of brain-behavior relationships and suggest that comparable personality scores are associated with similar brain states while people are engaged together in a social task.

## Data Availability

The raw DTW scores and additional data used in our previous research paper (Hinvest et al., 2025) is available via the Open Science Framework https://osf.io/6j95r/?view_only=7f77747ea53f42389898cfbbaa6481a. The DTW average and personality data from this project can be accessed by emailing the corresponding author.
