# Supplementary material for: Greater similarity of conscientiousness scores in dyads is associated with greater interpersonal neural synchrony while completing a goal-oriented task: a brief report
Source: Front Hum Neurosci. 2025 Nov 3;19:1622203. doi: 10.3389/fnhum.2025.1622203 (PMC12620442; doi:10.3389/fnhum.2025.1622203)
Supplement: Supplementary file 1 [file Supplementary_file_1.docx]

**Greater similarity of conscientiousness scores in dyads is associated with greater interpersonal neural synchrony while completing a goal-oriented task: A brief report - Supplementary Material**

**EEG Acquisition and Dynamic Time Warping Analysis**

***EEG acquisition and preprocessing***

EEG signals were collected using Emotiv EPOC 14-channel headsets (www.emotiv.com). These headsets were selected because they are wireless and lightweight, thereby enabling more naturalistic interaction between dyad members compared with conventional wired EEG systems. To minimise motion artefacts, participants were instructed to keep their heads as still as possible throughout the task. Prior validation work has shown that the EPOC provides sufficient measurement sensitivity and acceptable signal-to-noise ratios for research conducted in semi-naturalistic environments (Badcock et al., 2013; de Lissa et al., 2015; Hairston et al., 2014). Six dyads were excluded from subsequent analyses due to persistent channel failure across the recording period. A single PC was used to record from both headsets and a manual trigger provided time-locking for both recordings and transcription.

The headset contains 14 scalp electrodes positioned according to the international 10–20 system (AF3, AF4, F3, F4, F7, F8, FC5, FC6, P7, P8, T7, T8, O1, O2) and two reference sensors, one over each hemisphere. Signals were sampled at 128 Hz. A 5th-order sinc filter is applied online by the hardware, bandpassing the data between 0.16 and 43 Hz; therefore, no additional offline band-pass filtering was required.

Eye-movement artefacts were corrected in BESA Research 7.0 (Berg & Scherg, 1994) using its internal source-based topographic model of ocular activity. Thresholds were set at 150 μV for horizontal EOG and 250 μV for vertical EOG.

To further assess and refine data quality, an additional custom artefact-rejection routine was implemented. A data point was labelled as an outlier if both of the following criteria were met:

1. The smallest difference between the target point and its two immediate neighbours exceeded three times the difference between those neighbours; and
2. That same difference exceeded 20% of the inter-percentile range of the signal.

When both criteria were satisfied, the datapoint was removed. This procedure resulted in negligible data loss across participants (≤ 1.7% of datapoints from any one dataset, typically much less).

***Frequency decomposition***

Following artefact correction, signals were averaged across electrodes within each participant. Data were segmented into relevant epochs and decomposed into canonical EEG frequency bands (delta, theta, alpha, beta, gamma). Decomposition was carried out using a Butterworth filter (Proakis & Manolakis, 1992) implemented via the *butter* function in the *signal* package in R (Ligges et al., 2021). To isolate task-related activity, signals obtained during control conditions were subtracted from those recorded during social interaction. Exploratory regression analyses showed minimal divergence in results across frequency bands; therefore, all bands were pooled for subsequent analyses.

**Dynamic Time Warping (DTW)**

DTW analyses were conducted in R using the *dtw* package (Giorgino, 2022). As a reference signal, a 1000-timestep segment was randomly sampled from the baseline (non-interaction) period. This segment was concatenated with itself until it matched the duration of the corresponding conversational signal and then subtracted from the conversational signal to control for non-interaction baseline activity. Several steps were taken to improve DTW robustness:

- **Z-normalisation:** Each signal was transformed to mean = 0 and standard deviation = 1, ensuring comparability across participants and conditions(Rakthanmanon et al., 2013).
- **Endpoint trimming:** Because Butterworth filtering introduced variability at epoch boundaries, 10% was removed from each end prior to DTW.
- **Relaxed boundaries:** The “*open.end* = TRUE” argument in *dtw* was used, reducing the likelihood of poor local alignments by allowing non-strict endpoint matching (Giorgino, 2009).
- ***Window* constraint:** a *window* constraint was instituted to reduce the volume of required computation within the DTW matrix; here, Sakoe-Chiba band was chosen to determine the window’s boundaries as it gives the opportunity to each timeframe datum in a signal to be compared with all the data points, within the window, from the corresponding signal. Moreover, Sakoe-Chiba band is claimed to be superior, in terms of DTW performance, to Itakura Parallelogram constraint method (Geler et al., 2019).

Once alignment paths were computed, DTW scores were normalised to account for differences in epoch length and amplitude, using the following expression: Normalised DTW = DTW score / ((M + N) · |AUC1 – AUC2|)
where M = length of the first signal (width of the DTW matrix), N = length of the second signal (height of the DTW matrix), and AUC1/AUC2 = areas under the curve for each signal.

Normalised DTW values were then entered into linear mixed-effects models to test their relationship with linguistic markers of social identity across conversational stages.

**References**

Badcock, N.A., Mousikou, P., Mahajan, Y., de Lissa, P., Thie, J. and McArthur, G. (2013). Validation of the Emotiv EPOC® EEG gaming system for measuring research quality auditory ERPs. *PeerJ*, [online] 1. doi:https://doi.org/10.7717/peerj.38.

Berg, P. and Scherg, M. (1994). A multiple source approach to the correction of eye artifacts. *Electroencephalography and Clinical Neurophysiology*, 90(3), pp.229–241. doi:https://doi.org/10.1016/0013-4694(94)90094-9.

de Lissa, P., Sörensen, S., Badcock, N., Thie, J. and McArthur, G. (2015). Measuring the face-sensitive N170 with a gaming EEG system: A validation study. *Journal of Neuroscience Methods*, 253, pp.47–54. doi:https://doi.org/10.1016/j.jneumeth.2015.05.025.

Geler, Z., Kurbalija, V., Ivanovic, M., Radovanovic, M. and Dai, W. (2019). Dynamic time warping: Itakura vs Sakoe-Chiba. *2019 IEEE International Symposium on INnovations in Intelligent SysTems and Applications (INISTA)*. doi:https://doi.org/10.1109/inista.2019.8778300.

Giorgino, T. (2009). Computing and visualizing Dynamic Time Warping alignments in R: The dtw package. *Journal of Statistical Software*, 31(7). doi:https://doi.org/10.18637/jss.v031.i07.

Hairston, D.W., Whitaker, K.W., Ries, A.J., Vettel, J.M., Bradford, J.C., Kerick, S.E. and McDowell, K. (2014). Usability of four commercially-oriented EEG systems. *Journal of Neural Engineering*, 11(4), p.046018. doi:https://doi.org/10.1088/1741-2560/11/4/046018.

Proakis, J.G. and Manolakis, D.G. (1992). *Digital Signal Processing*. MacMillan Publishing Company.

Rakthanmanon, T., Campana, B., Mueen, A., Batista, G., Westover, B., Zhu, Q., Zakaria, J. and Keogh, E. (2013). Addressing big data time series. *ACM Transactions on Knowledge Discovery from Data*, 7(3), pp.1–31. doi:https://doi.org/10.1145/2500489.
